# Supplementary material for: Effects of using wearable devices to monitoring physical activity in pulmonary rehabilitation programs for chronic respiratory diseases: A systematic review protocol
Source: PLoS One. 2024 Jul 26;19(7):e0308109. doi: 10.1371/journal.pone.0308109 (PMC11280527; doi:10.1371/journal.pone.0308109)
Supplement: S1 File — (DOCX) [file pone.0308109.s002.docx]

**Terms used in the search strategy in Medline database**

#1 ("Pulmonary Disease, Chronic Obstructive"[Mesh]) OR ("Lung Diseases, Obstructive"[Mesh]) OR (Chronic Obstructive Lung Disease*) OR (Chronic Obstructive Pulmonary Disease*) OR (COAD) OR (COPD) OR (Chronic Obstructive Airway Disease*) OR (Airflow Obstruction*, Chronic) OR (Chronic Airflow Obstruction*)

#2 ("Asthma"[Mesh]) OR (Bronchial Asthma) OR (Asthma, Bronchial)

#3 #1 OR #2

#4 ("Exercise Therapy"[Mesh]) OR ("Exercise"[Mesh]) OR ("Resistance Training"[Mesh]) OR (Remedial Exercise*) OR (Exercise*, Remedial) OR (Therap*, Exercise) OR (Exercise Therapies) OR (Rehabilitation Exercise*) OR (Exercis*, Rehabilitation) OR (Pulmonary Rehabilitation) OR (Rehabilitation Program*) OR (Physical Fit) OR (Physical Train*) OR (Physical Activ*) OR (Exercise*, Aerobic) OR (Aerobic Exercise*) OR (Strength Training) OR (Training, Strength)

#5 ("Wearable Electronic Devices"[Mesh]) OR (Device, Wearable Electronic) OR (Electronic Device, Wearable) OR (Wearable Electronic Device*) OR (Wearable Technolog*) OR (Technology, Wearable) OR (Wearable Device*) OR (Device, Wearable) OR (Wearable Computer*) OR (Computer, Wearable) OR (Electronic Skin) OR (Skin, Electronic) OR (smartwatch*) OR (Physical Activity Monitor) OR (Physical Activity Feedback) OR (Activity Monitor) OR (Activity Feedback) OR (Step Count) OR (Pedometer*) OR (Actigraph*) OR (Acceleromet*)

#6 #3 AND #4 AND #5
