# Supplementary material for: Effects of using wearable devices to monitoring physical activity in pulmonary rehabilitation programs for chronic respiratory diseases: A systematic review protocol
Source: PLoS One. 2024 Jul 26;19(7):e0308109. doi: 10.1371/journal.pone.0308109 (PMC11280527; doi:10.1371/journal.pone.0308109)
Supplement: Attachment — Submitted filename: Response to Reviewers.docx [file pone.0308109.s003.docx]

Natal, RN, July 01, 2024

Dear Editors and reviewers,

We would like to express our sincere gratitude to you and the reviewers for the careful and insightful review of our manuscript titled “Effects of Using Wearable Devices to Monitoring Physical Activity in Pulmonary Rehabilitation Programs for Chronic Respiratory Diseases: A Systematic Review Protocol.” We appreciate the constructive feedback provided, which has significantly contributed to improving the quality and clarity of our work. Below, we address each of the points raised by the reviewers.

Journal Requirements:

1. The manuscript was formatted according to PLOS ONE style requirements, including those for file naming.
2. Was added to cover letter that: “ the progress of study has not started and will not begin until the article is accepted by the journal”.
3. We confirm that “All relevant data are within the manuscript and its Supporting Information files”. The data that will be used for the analyzes will be made available in supplementary material when submitting the complete work.

Reviewers’ comments:

1. In the case of articles written in languages the authors do not master, professional translation services will be hired to interpret and extract data from these articles.
2. Physical activity (eg. number of steps recorded by wearable devices or measured by self-reported physical activity questionnaires. For analysis purposes, this outcome will be standardized using the standard mean difference (SMD) in the meta-analysis. If a meta-analysis is not feasible, the difference between pre and post-intervention measurements will be calculated and the percentage of this difference will be computed, standardizing the data to a common unit of measurement).

Thank you once again for your time and consideration.

Sincerely,

Thaianne Rangel Agra Oliveira

(Department of Physiotherapy, Federal University of Rio Grande do Norte, Natal, RN, Brazil)
